# Domestic Correspondence

**Published:** 1888-10

**Authors:** B. M. Behrens

**Affiliations:** Chicago


### DOMESTIC CORRESPONDENCE.

CHICAGO, September 6, 1888.

*To the Editor of the Medical Journal and Examiner:*

Allow me to correct a misprint in the September number, that is liable to do more mischief than I would like to take the responsibility for if carried out by the otologists. Page 140, 7th line from below, has *inject* the internal wall, instead of *inspect*, etc.

While at it, I beg leave to make a few remarks about a passage on the same page. The article says: "After a discussion of the history of the procedure and a description of the various ways in which it has been employed," etc. It is needless to say that the procedure, as being entirely new, has no history; but what the *Journal* means to mention is, of course, the various operations that have been tried, and the history of which, I, in my paper, let pass review, to make the difference between these and my procedure conspicuous.

If I am allowed a little more space in your valuable paper, I would like to say but a few words more about my procedure. I presume it is understood by the otologists that this is to be regarded as an improvement of the sounding, at the same time, as by cutting through the drumhead the first and most important step is made towards operating in the middle ear, if need be. The principal advantage of this is that all the hearing bones can he reached with the instruments, while application of sound externally don't carry its influence further than to the malleus or malleus-incus joint at most, if there is an anchylosis of this joint. Once inside of the drumhead I can apply the sound or hook on manubrium, the long process of incus or incus-stapedio joint, and can hook stapes also, although, as will be seen in my paper, I am no advocate of using violence, sufficient force can be applied on the manubrium and incus-stapedio joint to bring about this slightly increased mobility of the joints that we just want, and nothing more. I regard it a gross mistake to ever forget what a delicate structure we here have to deal with.

Very respectfully,

B. M. BEHRENS.

